# Visual dysfunction in Parkinson’s disease

**DOI:** 10.1093/brain/aww175

**Published:** 2016-07-13

**Authors:** Rimona S. Weil, Anette E. Schrag, Jason D. Warren, Sebastian J. Crutch, Andrew J. Lees, Huw R. Morris

**Affiliations:** aww175-1 1 Institute of Neurology, University College London, London, UK; aww175-2 2 National Hospital for Neurology and Neurosurgery, Queen Square, London, UK; aww175-3 3 Department of Clinical Neurosciences, Royal Free Hospital NHS Trust, London, UK; aww175-4 4 Dementia Research Centre, UCL Institute of Neurology, University College London, London, UK

**Keywords:** vision, perception, Parkinson’s disease, cognition

## Abstract

Patients with Parkinson’s disease have a number of specific visual disturbances. These include changes in colour vision and contrast sensitivity and difficulties with complex visual tasks such as mental rotation and emotion recognition. We review changes in visual function at each stage of visual processing from retinal deficits, including contrast sensitivity and colour vision deficits to higher cortical processing impairments such as object and motion processing and neglect. We consider changes in visual function in patients with common Parkinson’s disease-associated genetic mutations including *GBA* and *LRRK2*. We discuss the association between visual deficits and clinical features of Parkinson’s disease such as rapid eye movement sleep behavioural disorder and the postural instability and gait disorder phenotype. We review the link between abnormal visual function and visual hallucinations, considering current models for mechanisms of visual hallucinations. Finally, we discuss the role of visuo-perceptual testing as a biomarker of disease and predictor of dementia in Parkinson’s disease.

## Teaser

Visual symptoms are frequently reported in Parkinson’s disease. Weil *et al.*. relate visual changes to underlying brain regions, and consider mechanisms for visual hallucinations. They examine links between visual changes and other features of Parkinson’s disease and discuss the role of visual dysfunction as a marker of dementia.

## Graphical abstract

**Figure:**
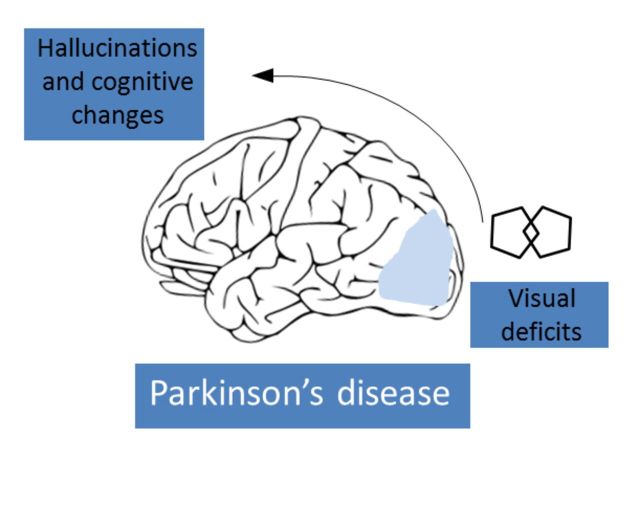
Visual symptoms are frequently reported in Parkinson’s disease. Weil *et al.*. relate visual changes to underlying brain regions, and consider mechanisms for visual hallucinations. They examine links between visual changes and other features of Parkinson’s disease and discuss the role of visual dysfunction as a marker of dementia.

## Introduction

The contribution of non-motor symptoms to a reduced quality of life in Parkinson’s disease is now widely recognized. In addition to complaints attributable to autonomic, gastrointestinal and cognitive dysfunction, visual symptoms are frequently reported (Davidsdottir *et al.*, 2005). Although many groups have demonstrated changes in visual perception in Parkinson’s disease, until now, most of these have focused on changes attributed to ophthalmic pathology. Here, we review the changes in visual perception in Parkinson’s disease under four separate sections. First we examine the evidence for visual deficits from early sensory discrimination to higher visual dysfunction. Second, we consider the neurobiology underlying visual changes including location of deficits and associated genetic mutations. Third, we examine the link between visual dysfunction and other clinical manifestations such as visual hallucinations and consider current theories of visual hallucinations. Finally we consider the role for testing visual perceptual function in early detection of dementia in Parkinson’s disease.

### The scope of the problem of vision in Parkinson’s disease

Patients with Parkinson’s disease frequently report problems with visual tasks, such as navigating around everyday environments and using maps (Bowen *et al.*, 1972; McDowell and Harris, 1997*b*; Davidsdottir *et al.*, 2005). In questionnaire studies, 78% of patients with Parkinson’s disease report at least one visual symptom, including difficulty reading, sometimes with double vision, and misjudging objects and distances (Archibald *et al.*, 2011; Urwyler *et al.*, 2014). Visual hallucinations are also common in Parkinson’s disease, with a reported prevalence as high as 74% after 20 years of disease (Fenelon *et al.*, 2000; Hely *et al.*, 2008). The underlying mechanisms are still not well understood, despite multiple proposed mechanisms (Barnes *et al.*, 2003; Collerton *et al.*, 2005; Diederich *et al.*, 2014; Shine *et al.*, 2014). A deeper understanding of changes in visual perception in Parkinson’s disease will greatly enhance our ability to manage visual hallucinations. Visuo-perceptual problems are well described in dementia with Lewy bodies and in Parkinson’s disease dementia, but there is growing evidence pointing to changes in visual processing earlier in the disease course. Here we examine changes in visual processing that are seen in patients with Parkinson’s disease and no other cognitive deficits. The studies cited here involve patients in the mid-stages of Parkinson’s disease at an average Hoehn and Yahr stage of 2 while on their regular Parkinson’s drugs, unless stated otherwise.

## Evidence for changes in visual processing

### Ophthalmic visual processing: visual acuity

**Figure 1:**
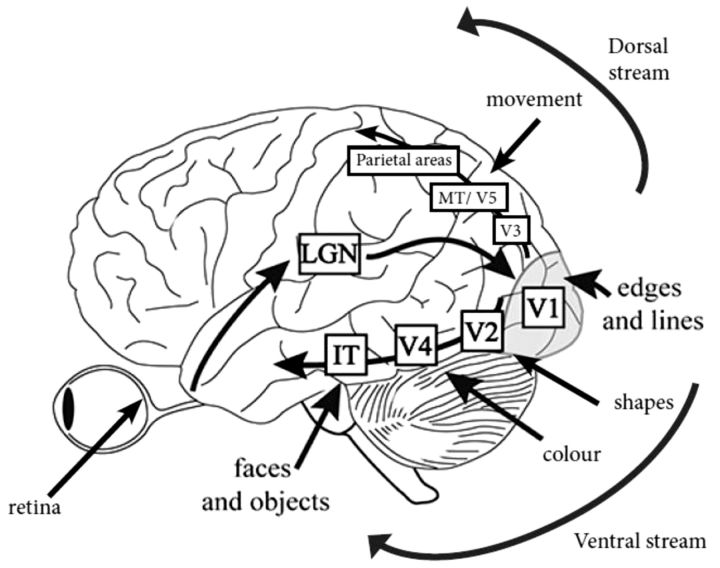
**Functional anatomy of healthy human vision.** Information passes from the retina, via the optic nerve and optic tract to the lateral geniculate nucleus (LGN) in the thalamus. From there, signals project via the optic radiation to the primary visual cortex (V1). Cells in V1 process simple local attributes such as the orientation of lines and edges. From primary visual cortex, information is organized as two parallel hierarchical processing streams: the ventral stream identifies features of objects and passes from V1 through areas V2 and V4 to the inferior temporal cortex. The dorsal stream processes spatial relations between objects and projects through areas V2 and V3 to the superior temporal and parietal cortices. Almost all connections between regions are reciprocal, with further feedback projections from areas outside this pathway. IT = infero-temporal region; MT/V5 = motion processing region. Adapted from Manassi *et al.* (2013) with permission (copyright held by the Association for Research in Vision and Ophthalmology).

Visual acuity is the ability to resolve the details of a stimulus and is affected by ophthalmic factors, including retinal deficits, rather than cortical disease. See Fig. 1 for an overview of the functional anatomy of healthy vision. Visual acuity may be impaired in Parkinson’s disease (Jones *et al.*, 1992; Archibald *et al.*, 2011), and this effect is not corrected by dopamine. Higher scores on parkinsonian motor examination may predict poor visual acuity (Archibald *et al.*, 2011). However, the differences are small (Jones *et al.*, 1992) and are not seen in early stage untreated patients (Biousse *et al.*, 2004) or in some studies involving mid-stage patients (Regan and Maxner, 1987), although in many studies acuity is regarded as normal if it is 6/9 or better (Bodis-Wollner *et al.*, 1987) or is considered on a case-by-case basis, which may miss subtle group differences in acuity.

### Contrast sensitivity

**Figure 2:**
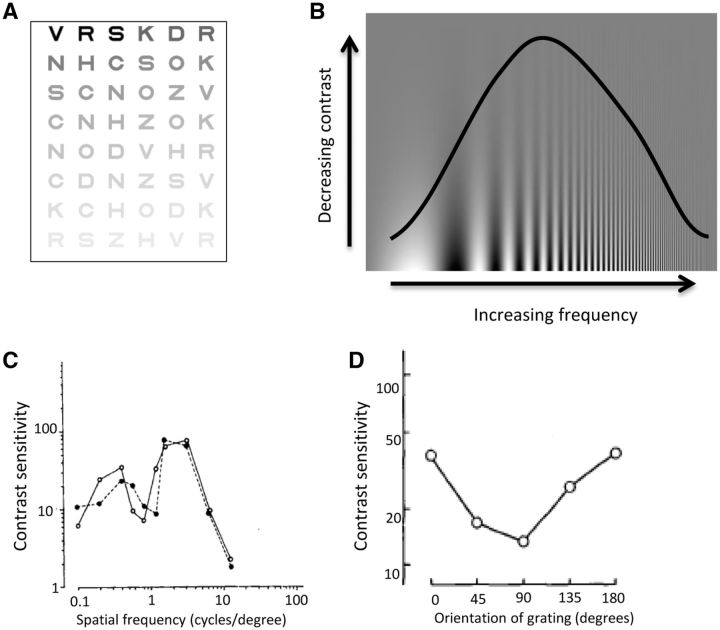
**Measuring visual function: visual contrast sensitivity.** (**A**) Pelli-Robson Chart for measuring contrast sensitivity. Letters are of the same size but reducing contrast (Pelli *et al.*, 1988). (**B**) A Campbell-Robson grating, showing increasing spatial frequency plotted against decreasing contrast. Visibility is indicated by the inverted U-shaped curve, with maximal visibility at mid-range spatial frequency, although individuals vary in their precise point of visibility (Campbell and Robson, 1968). (**C**) Loss of contrast sensitivity at the middle range of spatial frequencies in Parkinson’s disease. Example contrast sensitivity curve for a patient with Parkinson’s disease (each line represents one eye). Adapted from Bulens *et al.* (1986) with permission. (**D**) Effect of orientation of visual grating on contrast sensitivity in Parkinson’s disease. Contrast sensitivity for perception of gratings at orientations ranging from vertical (0 degrees), through horizontal (90 degrees) and back to vertical (180 degrees). Reduced contrast sensitivity is seen for horizontal gratings. Adapted from Regan and Maxner (1987) with permission.

Contrast sensitivity is the ability to discriminate an object from its background and is affected by lesions in the eye (including retina) as well as in thalamic or cortical locations. In tests using vision charts with letters of the same size but reducing contrast, patients with Parkinson’s disease have normal acuity but impaired contrast detection (Regan and Neima, 1984) (Fig. 2A and B). With more quantitative measurements, patients with Parkinson’s disease show loss of contrast sensitivity across a range of spatial frequencies and in both foveal (central) and peripheral locations (Silva *et al.*, 2005). Contrast sensitivity loss is partly reversible with l-DOPA treatment, in both early (Bulens *et al.*, 1987) and mid-stage Parkinson’s disease (Bodis-Wollner *et al.*, 1987) but it eventually deteriorates again as the disease advances (Diederich *et al.*, 2002).

### Colour vision

In the healthy brain, colour is processed by photoreceptor cones in the retina and at higher levels from primary visual cortex (V1) to extra-striate visual cortex (V4). Disordered colour discrimination is recognized in Parkinson’s disease (Price *et al.*, 1992; Buttner *et al.*, 1995), even in the early stages of the disease, in untreated patients (Buttner *et al.*, 1995). Abnormalities in colour vision have also been correlated with axial motor symptoms (Oh *et al.*, 2011), with motor speed (in untreated patients) (Muller *et al.*, 1999) and with disease duration (Price *et al.*, 1992). Colour vision deficits progress over time in both treated and untreated patients (Diederich *et al.*, 2002; Muller *et al.*, 2002). Colour discrimination may be confounded by cognitive or motor deficits in some studies (Regan *et al.*, 1998), due to the length of time needed to administer the widely used Farnsworth-Munsell 100 Hue test. This test requires patients to arrange 100 coloured discs in sequence. Colour discrimination, as measured in this way, correlates with cognitive performance and with white matter changes in posterior brain structures (Bertrand *et al.*, 2012). However, abnormalities in colour vision can also be found using tests that are less susceptible to these confounds (Silva *et al.*, 2005). Colour loss, when measured in this way, is distinct from normal ageing and seems to particularly affect the protan and deutan (red–green) axes (Silva *et al.*, 2005), although other investigators have found greatest deficits in the tritan (blue–yellow) axis (Haug *et al.*, 1995; Regan *et al.*, 1998). It is unclear whether colour vision abnormalities in Parkinson’s disease reflect retinal or cortical disease, but given that differences can be seen along several axes (Silva *et al.*, 2005), it is likely that changes in colour sensitivity are multifactorial.

### Are visual abnormalities in Parkinson’s disease due to retinal dysfunction?

Deficits in visual acuity, contrast and colour in Parkinson’s disease are at least partly due to retinal dopamine deficiency. The healthy human retina contains dopaminergic amacrine cells (Frederick *et al.*, 1982) and patients with Parkinson’s disease have reduced dopamine innervation around the fovea (Nguyen-Legros, 1988). Autopsy studies show decreased retinal dopamine concentrations in Parkinson’s disease (Harnois and Di, 1990). The inner retinal layer is thinner in patients with Parkinson’s disease, measured using optical coherence tomography (Hajee *et al.*, 2009). Retinal electrical activity is decreased compared to healthy controls, measured by electroretinogram (Tagliati *et al.*, 1996; Moschos *et al.*, 2011) and improves with l-DOPA treatment (Peppe *et al.*, 1995). Intriguingly, patients with Parkinson’s disease have shorter durations of negative afterimages than healthy controls (Khadjevand *et al.*, 2010), a process thought to be mediated in health by retinal dopaminergic neuromodulation (Wink and Harris, 2000). In some patients, contrast sensitivity thresholds and visual evoked potential latencies differ between eyes (Bulens *et al.*, 1986, 1987; Bodis-Wollner *et al.*, 1987), raising the likelihood of prechiasmal involvement. The recent finding in the inner retinal layer of misfolded α-synuclein (Bodis-Wollner *et al.*, 2014*a*) and of phosphorylated α-synuclein (Beach *et al.*, 2014), the pathological hallmark of Parkinson’s disease, is striking evidence of retinal involvement in Parkinson’s disease. At least some aspects of visual dysfunction in Parkinson’s disease therefore involve disruption of processing at the level of the retina.

Some untreated patients also show loss of contrast sensitivity at specific intermediate spatial frequencies (Fig. 2C) (Bulens *et al.*, 1986). This is more suggestive of a cortical rather than retinal or geniculate site of disruption as cortical neurons show stronger selectivity for different spatial frequencies than neurons in the retina or thalamus (Campbell and Robson, 1968; De Valois *et al.*, 1982; Regan, 1982). In addition to spatial frequency selectivity, contrast sensitivity in Parkinson’s disease is often orientation-specific, with greatest deficits for horizontal gratings (Regan and Maxner, 1987; Bulens *et al.*, 1988; Trick *et al.*, 1994) (Fig. 2D). As receptive fields of neurons in primary visual cortex are tuned to specific orientations (Hubel *et al.*, 1978), these findings implicate primary visual cortex as the locus for deficits in contrast sensitivity in Parkinson’s disease, rather than just the retina or the thalamus, which are not linked to orientation specificity.

### Changes in eye movements

Subtle oculomotor changes can be seen in Parkinson’s disease, with voluntary saccades particularly affected. Latency and velocity are preserved but amplitude is reduced, producing hypometric movements (Ventre *et al.*, 1992; Kimmig *et al.*, 2002; MacAskill *et al.*, 2002). Saccades to a remembered target are particularly impaired and show a multistep pattern (Crawford *et al.*, 1989). These are thought to arise from deficits in oculomotor pathways in the brainstem, cerebellum, basal ganglia and frontal lobes. In addition, patients with Parkinson’s disease commonly show convergence insufficiency, that impacts on near activities and may lead to double vision on reading (Almer *et al.*, 2012; Nowacka *et al.*, 2014). Pronounced oculomotor abnormalities, when present, usually indicate an atypical Parkinsonian syndrome, but supranuclear vertical gaze impairment has rarely been reported in pathologically confirmed Parkinson’s disease. Early decreased saccadic velocity, preservation of saccadic latency and paresis of vertical saccades favour a diagnosis of progressive supranuclear gaze palsy (Bhidayasiri *et al.*, 2001). Cerebellar type eye movement abnormalities, such as gaze-evoked nystagmus, abnormal vestibulo-ocular-reflex suppression, down-beat nystagmus, and excessive square wave jerks are more suggestive of multiple system atrophy (for a review see Pinkhardt and Kassubek, 2011).

### Visual processing in early cortical visual areas: line orientation, pattern perception and depth perception

The earliest stages of cortical visual processing involve discrimination of local attributes. Neurons in primary visual cortex (V1) are sensitive to the orientation of lines and edges (Fig. 1) and changes in visual acuity and contrast sensitivity in patients with Parkinson’s disease, may be caused by deficits in early cortical visual processing. In tests comparing the ability to distinguish between lines of differing directions (Benton *et al.*, 1978), several studies showed impaired performance in patients with Parkinson’s disease (Montse *et al.*, 2001; Uc *et al.*, 2005; Gullett *et al.*, 2013) that correlated with duration and severity of disease. Although primary visual cortex is sensitive to changes in line orientation, judgements of matching lines may also involve higher order visuospatial processing. Indeed, in lesion studies of non-parkinsonian patients, failure in orientation judgements is most strongly associated with parietal lobe lesions (Tranel *et al.*, 2009).

Pattern perception and figure ground discrimination involve a combination of earlier visual cortical processing and higher level interpretation. In tasks requiring completion of patterns, patients with Parkinson’s disease perform worse than healthy controls (Flowers and Robertson, 1995). Similarly, patients performing a figure–ground discrimination task (identifying figures embedded in more complex figures) made more errors than healthy controls (especially those with more advanced disease) (Flowers and Robertson, 1995). In neuroimaging studies, patients with worse performance in similar tasks also have reduced grey matter density in their superior parietal lobes (Pereira *et al.*, 2009).

Depth perception is also affected in patients with Parkinson’s disease and is associated with worse motor dexterity and decreased colour perception (Sun *et al.*, 2014). Untreated Parkinson’s patients with impaired depth perception have been shown, in neuroimaging studies, to have reduced grey matter volume in visual association areas (Koh *et al.*, 2013).

### Peripheral vision

Information from peripheral visual fields is processed at multiple levels from the retina to higher cortical regions. When patients are given a visual task, and presented with distracting images in their peripheral fields at the same time, the irrelevant peripheral objects interfere with the main task to a much greater extent than they do for healthy volunteers (McDowell and Harris, 1997*a*). Conversely, although patients with Parkinson’s disease are more distracted by targets shown in the periphery, they have greater difficulty discriminating details of peripheral images and perceive these images less strongly than healthy volunteers (Harris *et al.*, 1992; Sampaio *et al.*, 2011). A combination of reduced contrast sensitivity in the periphery with greater distraction from peripheral objects may provide a perceptual explanation for the common phenomenon of freezing in doorways and crowded surroundings seen in the later stages of Parkinson’s disease in many patients (McDowell and Harris, 1997*a*).

### Object perception

In the healthy brain, object processing occurs in V2, V4 and the lateral occipital complex, especially in the right hemisphere (Grill-Spector *et al.*, 1999; Kourtzi and Kanwisher, 2000; Large *et al.*, 2007). Patients with early stage Parkinson’s disease are as good at recognizing common objects from silhouettes as healthy controls (Hipp *et al.*, 2014). However, by mid-stage, they start to have difficulties in identifying overlapping objects (Pillon *et al.*, 1989; Mosimann *et al.*, 2004), with worse performance occurring in patients with left-sided motor symptoms (Levin *et al.*, 1991). This is consistent with right hemisphere predominance in object recognition and suggests asymmetry in cortical as well as nigral involvement.

Mental rotation is another aspect of object recognition that may be affected in Parkinson’s disease. In a previous small study, patients with Parkinson’s disease made more errors when matching objects in 3D space (Lee *et al.*, 1998). Greater differences were seen at larger angular discrepancies, when objects were more rotated, suggesting that the abnormality is caused by changes in the mental ability to rotate objects, rather than simply due to differences in pattern matching. Although error rates were not increased when objects were rotated in 2D space, responses were slower, suggesting some impaired performance for 2D rotation. A recent large study found impaired performance in 2D rotation in patients with early stage Parkinson’s disease, with even greater impairments in patients carrying the H1 *MAPT* haplotype (microtubule-associated protein tau), particularly in more difficult forms of the task (Nombela *et al.*, 2014) (see below for further discussion on genetic associations).

### Visuospatial construction

Patients with Parkinson’s disease make more errors when copying and recalling complex figures than healthy controls (Ogden *et al.*, 1990; Uc *et al.*, 2005). Copying intersecting pentagons is frequently used as a simple bedside measure of visuospatial function in Parkinson’s disease. It is quick to perform and is a component of the Mini-Mental State Examination (Williams-Gray *et al.*, 2013; Lawson *et al.*, 2014). Longitudinal studies show that it has value in predicting the development of dementia in Parkinson’s disease. In a recent large cross-sectional study, patients with Parkinson’s disease showed slightly lower scores for copying intersecting pentagons than healthy controls (Garcia-Diaz *et al.*, 2014), with a correlation between performance in pentagon copying and reduced cortical thickness in parieto-temporal regions. However, no difference was seen by another group using a less detailed scoring system and fewer patients (Filoteo *et al.*, 2014). This highlights the relatively low sensitivity of this test in assessing visuospatial performance. In clock drawing tests, patients with Parkinson’s disease (and no dementia) make more errors than healthy controls (Dal *et al.*, 1989; Stella *et al.*, 2007; Saur *et al.*, 2012) and errors in drawing houses are also reported in Parkinson’s disease (Kulkarni *et al.*, 2013). However, all these tests lack specificity for visuospatial function as they involve several cognitive domains in addition to visuospatial processing, including praxis, memory and executive function.

### Motion perception

Parkinson’s disease impairs the perception of motion, which is strongly linked with the middle temporal area MT/V5 (Zeki *et al.*, 1991; Tootell *et al.*, 1995) (Fig. 1). Patients have greater difficulty in detecting a moving grating than a static one, which is a reversal of the normal pattern where motion of a grating is detected before it can be identified (Mestre *et al.*, 1990; Haug *et al.*, 1994). Patients with Parkinson’s disease also have higher thresholds than healthy controls for detecting the direction of motion (Trick *et al.*, 1994), although in a speed discrimination task, patients with Parkinson’s disease performed no worse than controls (Mosimann *et al.*, 2004). Finally, in a structure from motion task, where volunteers detect an object that is formed from the contrast of moving dots, patients with Parkinson’s disease show higher thresholds for correctly identifying objects (Uc *et al.*, 2005).

### Spatial neglect

Patients with Parkinson’s disease predominantly affecting their left side show a rightward bias when bisecting a horizontal line. (Lee *et al.*, 2001*b*; Laudate *et al.*, 2013). This reflects spatial neglect on the left, ipsilateral to the side affected by motor symptoms, and suggests involvement of right parietal cortex. Line bisection bias is not seen in patients with right-sided Parkinson’s disease. Similarly, in line cancellation tasks, patients with left-sided (but not right-sided) Parkinson’s disease show impaired line cancellation (Villardita *et al.*, 1983). In a related phenomenon, patients with left sided Parkinson’s disease perceive objects within their left visual field as smaller than on the right (Harris *et al.*, 2003) and experience a reduced visual representation of doorways (Lee *et al.*, 2001*a*). This perceptual difference may contribute mechanistically to the phenomenon of freezing in doorways. In eye movement analyses, when searching for a target, patients with left-sided Parkinson’s disease will explore the right hemifield first, in contrast with healthy controls and patients with right-sided Parkinson’s disease, who show a leftward bias (Ebersbach *et al.*, 1996). Similarly, patients with left-sided Parkinson’s disease show longer latencies for saccades to left-sided targets compared to the right (Ventre *et al.*, 1992). Whether differences in spatial representation are caused by eye movement abnormalities, or eye movements reflect the differences in perception could be tested in future studies.

### Face and emotion recognition

The ability to recognize faces is impaired in Parkinson’s disease (Levin *et al.*, 1991), with performance correlated with grey matter density in the fusiform face area, the region involved in face recognition in the heathy brain (Pereira *et al.*, 2009). There is particular difficulty in interpreting facial expressions (Sprengelmeyer *et al.*, 2003), with greater impairment for negative emotions including disgust (Suzuki *et al.*, 2006), sadness (Hipp *et al.*, 2014) and fear (Jacobs *et al.*, 1995; Dujardin *et al.*, 2004; Clark *et al.*, 2008; Gray and Tickle-Degnen, 2010). This greater deficit for emotional face processing in Parkinson’s disease may be explained by the presence of two distinct face processing pathways that are sensitive to different spatial frequency ranges: face identification, associated with the fusiform face area, is more activated by high spatial frequency ranges (detailed visual information). In contrast, subcortical pathways, including the amygdala, are activated by fearful faces, and are driven by coarse, low spatial frequency information (Vuilleumier *et al.*, 2003). The finding that performance improves in patients treated with l-DOPA is consistent with the involvement of dopaminergic neurons in this pathway (Sprengelmeyer *et al.*, 2003).

## Neurobiology underlying visual changes: genetic basis for heterogeneity

Important insights into the heterogeneity of Parkinson’s disease have emerged with the discovery in recent years of genetic mutations that cause or predispose to Parkinson’s disease, some of which are associated with defined molecular pathways. Visual perceptual dysfunction associated with specific mutations may therefore implicate potential pathophysiological mechanisms. Patients carrying *LRRK2* mutations have better colour discrimination (Marras *et al.*, 2011) and fewer cognitive deficits than patients with idiopathic Parkinson’s disease (Alcalay *et al.*, 2013) (Table 1). Parkinson’s patients who carry *PARK2* mutations, which are associated with dysfunction in the mitochondrial system and have a more restricted distribution of neuropathology, perform better in cognitive tests than patients with sporadic Parkinson’s disease (Alcalay *et al.*, 2014).

**Table 1:** Associations between features linked with visual dysfunction and common Parkinson’s disease-associated genetic mutations

Potential pathway	Gene	Performance compared with idiopathic Parkinson’s disease		Frequency compared with idiopathic Parkinson’s disease	
		Colour vision	Cognitive performance	Visual hallucinations	RBD
Multiple implicated	*LRRK2*	Increased^a^	Increased^b^	Decreased^c^	Decreased^d^
Mitochondrial	*PARK2*	Increased^e^	Increased^f^	Not known	Similar^g^
Lysosomal	*GBA*	Possibly increased^h^	Decreased^i^	Increased^j^	Increased^k^
Lysosomal	*SNCA*	Not known	Decreased^l^^,^^m^	Increased^l^^,^^m^	Increased^l^^,^^n^

^a^Marras *et al.*, 2011; ^b^Alcalay *et al.*, 2013; ^c^Somme *et al.*, 2015; ^d^Ehrminger *et al.*, 2015; ^e^Kertelge *et al.*, 2010; ^f^Alcalay *et al.*, 2014; ^g^Sixel-Doring *et al.*, 2015; ^h^Simon-Tov *et al.*, 2015; ^i^Alcalay *et al.*, 2012; ^j^Neumann *et al.*, 2009; ^k^Gan-Or *et al.*, 2015; ^l^Konno *et al.*, 2016; ^m^Petrucci *et al.*, 2016; ^n^Nishioka *et al.*, 2009.

Conversely, patients with Parkinson’s disease that carry mutations in the gene encoding the lysosomal enzyme glucocerebrosidase (GBA) show deficits in visuospatial tasks (Alcalay *et al.*, 2012), have impaired visual memory (Zokaei *et al.*, 2014) and show a higher frequency of visual hallucinations (Neumann *et al.*, 2009; Wang *et al.*, 2014) than patients with idiopathic Parkinson’s disease. Intriguingly, patients with *GBA* mutations have decreased cerebral blood flow in the parietal lobe and precuneus (Goker-Alpan *et al.*, 2012), regions involved in visual perception (Cavanna and Trimble, 2006) and in visual memory (Kochan *et al.*, 2011). Patients with Parkinson’s disease that carry *GBA* mutations also show a higher frequency of clinical features that are often associated with visual dysfunction in Parkinson’s disease: they have a higher risk of cognitive deficits (Neumann *et al.*, 2009; Brockmann *et al.*, 2011; Winder-Rhodes *et al.*, 2013) and rapid eye movement (REM) sleep disorder than patients with idiopathic Parkinson’s disease (Beavan *et al.*, 2015; Gan-Or *et al.*, 2015) (see below for further discussion of clinical features associated with visual dysfunction in Parkinson’s disease).

Patients with Parkinson’s disease carrying mutations in the gene for synuclein, *SNCA*, also show higher rates of cognitive deficits, REM sleep behaviour disorder (RBD) and hallucinations (Nishioka *et al.*, 2009; Konno *et al.*, 2016; Petrucci *et al.*, 2016). Neuropathological studies of these patients show a greater α-synuclein burden in the cerebral cortex than in idiopathic Parkinson’s disease (Ikeuchi *et al.*, 2008; Obi *et al.*, 2008) and fluorodeoxyglucose (FDG) signal is reduced in the occipital lobes (visual processing regions) of patients carrying *SNCA* mutations compared with controls (Nishioka *et al.*, 2009). Both the *GBA* and *SNCA* genes are linked with the lysosomal pathway (Mazzulli *et al.*, 2011), suggesting the visual deficits associated with these genes may share a pathophysiological mechanism, with lysosomal dysfunction associated with cortical visual dysfunction.

Genetic polymorphisms may also play a role in predisposition to cognitive impairment in Parkinson’s disease. A common polymorphism in *MAPT* has been linked to dementia in Parkinson’s disease (Goris *et al.*, 2007) and patients carrying the H1 *MAPT* polymorphism make more errors in difficult spatial rotation tasks, and show reduced parietal cortex activity (Nombela *et al.*, 2014). The underlying mechanism for these findings may relate to differences in the cortical expression of 4- versus 3-repeat isoforms of tau (Williams-Gray *et al.*, 2009). Greater understanding of these genetic differences between individuals will be crucial in future studies seeking to define the explanations for the diversity in visuospatial function found in Parkinson’s disease

## Clinical relevance

### Clustering of symptoms with visual dysfunction in Parkinson’s disease

There is an increasing acknowledgement of the clinical heterogeneity of idiopathic Parkinson’s disease (Selikhova *et al.*, 2009; Halliday *et al.*, 2011; Sieber *et al.*, 2014) and differences in survival rates have been reported between clinical subtypes defined in longitudinal analysis (de Lau *et al.*, 2014). Visual dysfunction frequently co-exists with cognitive impairment, visual hallucinations, postural instability with gait disorder and RBD (Davidsdottir *et al.*, 2005; Marques *et al.*, 2010). Two distinct neuropsychological syndromes have been proposed in Parkinson’s disease: a frontal-striatal dopamine-mediated dysexecutive syndrome that does not progress to dementia; and a second form with prominent visuospatial and sematic fluency impairments that is more frequently associated with decline to dementia. Therefore, recognizing visuospatial impairment in the context of cognitive deterioration may have importance as a prognostic marker for dementia in Parkinson’s disease. (Williams-Gray *et al.*, 2009; Kehagia *et al.*, 2010).

### Relation with sleep abnormalities

Idiopathic RBD, characterized by loss of normal atonia during REM sleep, is considered a risk factor for Parkinson’s disease and other synucleinopathies: over 80% of idiopathic RBD patients may eventually develop a neurodegenerative disorder linked to α-synuclein accumulation in the brain (Iranzo *et al.*, 2013; Schenck *et al.*, 2013). Visuo-perceptual deficits have been reported in patients with RBD (who have not yet developed Parkinson’s disease), with impairments in colour vision and visuospatial construction (Boeve *et al.*, 1998; Ferini-Strambi *et al.*, 2004; Postuma *et al.*, 2009; Manni *et al.*, 2013). Those RBD patients with sensory abnormalities at baseline (including colour vision defects) have also been claimed to develop a form of Parkinson’s disease with more prominent cognitive involvement (Postuma *et al.*, 2011). Later, at mid-stage, this same association, RBD and poor colour discrimination, is again linked with a more rapid and aggressive disease course (Fereshtehnejad *et al.*, 2015). Although RBD is a rare prodromal sign in patients presenting with motor symptoms, up to a third of patients develop RBD in the course of their illness (Gagnon *et al.*, 2002; Manni *et al.*, 2010). When it occurs, it is associated with visuo-perceptual dysfunction, including errors in object recognition (Marques *et al.*, 2010) and complex figure copying (Vendette *et al.*, 2007). The presence of RBD in Parkinson’s disease is also predictive of progressive cognitive dysfunction (Sinforiani *et al.*, 2008; Postuma *et al.*, 2012; Nomura *et al.*, 2013).

The recent discovery of a novel photoreceptor system in the retina may provide a further link between sleep and visual dysfunction in Parkinson’s disease. A subset of retinal ganglion cells, known as melanopsin photoreceptors, are believed to play a role in regulating circadian rhythms. Dysfunction of these retinal ganglion cells, possibly by α-synuclein deposition, or by a change in dopamine levels, causes unopposed melatonin production, with subsequent effects on sleep (for a review see Schmoll *et al.*, 2011). Furthermore, the projection of these cells to brain regions involved in circadian and sleep functions as well as to visual areas such as the lateral geniculate nucleus may explain some of the daytime-dependent (not just luminance-dependent) visual symptoms of Parkinson’s disease (La Morgia *et al.*, 2011).

### Gait, postural instability and tremor

Visual perceptual deficits are frequently associated with the postural instability and gait disorder phenotype (PIGD). Visual abnormalities are more common in patients with freezing of gait (Davidsdottir *et al.*, 2005) and correlate with the severity of gait impairment (Uc *et al.*, 2005). Furthermore, visual contrast sensitivity predicts severity of freezing, independent of duration or severity of disease (Davidsdottir *et al.*, 2005). In patients with freezing of gait, step length reduces on going through narrow doorways, suggesting a perceptual mechanism interfering with movement planning (Almeida and Lebold, 2010; Cowie *et al.*, 2010).

Conversely, the tremor predominant phenotype is less associated with visual deficits. In questionnaire studies, patients without tremor report worse visual function (Seichepine *et al.*, 2011). Patients with increased tremor perform better on tasks of line orientation, mental reconstruction and rotation tasks (Levin *et al.*, 1991) and in longitudinal follow-up, presence of tremor is associated with a more benign course and fewer colour vision abnormalities (Fereshtehnejad *et al.*, 2015).

The link between visual perceptual deficits and gait abnormalities may be partly explained by the critical role of vision in balance (Day and Guerraz, 2007). Patients with Parkinson’s disease show a higher dependence on visual information for motor and postural control (Bronstein *et al.*, 1990). This may have a therapeutic role as it has been shown that visual cueing can improve walking in Parkinson’s disease (Stern *et al.*, 1980; Azulay *et al.*, 1999; Lewis *et al.*, 2000). However, the association of visual deficits with the akinetic rigid phenotype and relative sparing of visual dysfunction in the tremor predominant phenotype may also reflect distinct pathophysiological patterns of disease.

### Hallucinations: links with visuo-perceptual deficits

Visual hallucinations are common in Parkinson’s disease, with a prevalence of 30% (Holroyd *et al.*, 2001). Their presence is believed to be highly specific for Lewy body pathology (Williams *et al.*, 2008), and can be helpful in distinguishing between Parkinson’s disease and other parkinsonian syndromes such as multiple system atrophy (MSA-P) and progressive supranuclear palsy (PSP-P) (Williams and Lees, 2005). Visual hallucinations often occur in poor lighting and are associated with reduced visual acuity (Holroyd *et al.*, 2001; Matsui *et al.*, 2006; Archibald *et al.*, 2011) but can also be seen in patients with normal visual acuity (Diederich *et al.*, 1998; Biousse *et al.*, 2004). They are also associated with impaired contrast sensitivity and colour vision (Diederich *et al.*, 1998) and patients with visual hallucinations show greater deficits in object and face identification compared to those without hallucinations (Barnes *et al.*, 2003; Ramirez-Ruiz *et al.*, 2006; Gallagher *et al.*, 2011), reflecting involvement of higher cortical visual dysfunction. Recent reports of minor visual hallucinations in patients at the earliest stages of Parkinson’s disease (Pagonabarraga *et al.*, 2016), including in untreated patients, lends further support for changes in cortical visual processing even at the initial stages of Parkinson’s disease.

### Mechanistic considerations of minor hallucinations

‘Passage hallucinations’ are illusions of objects, commonly insects and small animals, moving across the peripheries of vision (Fenelon *et al.*, 2000). The combination of being more sensitive to irrelevant peripheral objects (McDowell and Harris, 1997*a*), and less able to discriminate details in the periphery could explain this phenomenon. Misinterpretations, or illusions, where innocuous objects such as piles of clothes are misidentified as animals, are also commonly reported in Parkinson’s disease. These may be generated by similar mechanisms of increased sensitivity but reduced discriminatory ability for objects in peripheral vision.

The phenomenon of blindsight might also help to explain minor illusions in Parkinson’s disease (Diederich *et al.*, 2014). Blindsight is the syndrome of retained visual abilities in patients with bilateral occipital damage who can accurately guess locations of objects in space despite the absence of conscious visual perception. It is mediated via alternate visual pathways from the retina, involving the thalamus, colliculus and amygdala and is a form of unconscious processing of visual information. It has been suggested that these alternate pathways are damaged in Parkinson’s disease, rendering patients ‘blind to blindsight’ (Diederich *et al.*, 2014). According to the model, patients with Parkinson’s disease have difficulty guessing locations in peripheral space, which may lead to the erroneous experience of objects in the lateral field. This conceptual framework may explain minor illusions but it cannot account for complex visual hallucinations that arise in the absence of any stimulus and at the point of fixation.

### Mechanistic considerations of complex hallucinations

The complex visual hallucinations reported in Parkinson’s disease almost invariably involve animate objects, are often at fixation and are more frequently experienced in dim light. They are usually non-frightening and occasionally enjoyed, but become increasingly distressing over time (for reviews see Fenelon *et al.*, 2000; Diederich *et al.*, 2009; Collerton *et al.*, 2015). Previous models to explain these hallucinations include cortical irritation caused by electrical over-activity in brain regions containing images (Levine and Finklestein, 1982). However, there is no evidence for cortical irritation in Parkinson’s disease and complex hallucinations are not strongly associated with diseases where cortical irritation is a feature, such as epilepsy.

A more attractive model considers hallucinations as ‘cortical release’ phenomena (Ffytche and Howard, 1999). In this model, that has also been used to explain the hallucinations seen in Charles Bonnet syndrome, stimulus-driven bottom-up visual processing is inhibitory in nature and when absent, this releases spontaneous activity. Visual hallucinations therefore arise from a lack of sensory input that leads to the release of stored images. However, loss of visual input alone, from eye disease or stroke, only infrequently leads to visual hallucinations and the visual deficits seen in Parkinson’s disease are not profound enough to cause absent sensory input.

Collerton and colleagues (2005) have proposed a Perception and Attention Deficit model based on proto-objects that are part of normal scene perception. These are object templates from higher visual processing regions projected to lower visual regions during normal scene perception. In health, visual input activates several competing proto-objects but attentional binding allows only one object to be perceived. During hallucinations, impaired sensory input causes activation of an incorrect proto-object. This, combined with impaired attentional binding, causes it to be incorrectly projected onto the intact visual scene. Although the combination of early perceptual deficits with higher attentional networks is likely to be important in generating visual hallucinations, this model has limitations. It cannot adequately explain the appearance of incongruent objects during hallucinations. It also implies that properly perceived objects should displace hallucinatory objects, causing the hallucination to disappear, which does not occur [see commentary by Morrison and David (2005) on Collerton *et al.*, 2005)].

The Activation-Input-Modulation model proposed by Hobson, is another hypothesis (Hobson *et al.*, 2000; Diederich *et al.*, 2005). The first dimension, activation, relates to level of alertness; the second is input, or sensory information. The third dimension, modulation, is the integration of the first two dimensions over time and invokes aminergic and cholinergic transmission. According to this model, movement along each dimension increases the likelihood for visual hallucinations. In this way, aberrant visual processing increases the propensity for visual hallucinations, as would changes in arousal levels. The modulation dimension is affected by pharmacological influences. One weakness of the model is that it fails to explain how visual hallucinations that are initially benign can become increasingly distressing with loss of insight.

**Figure 3:**
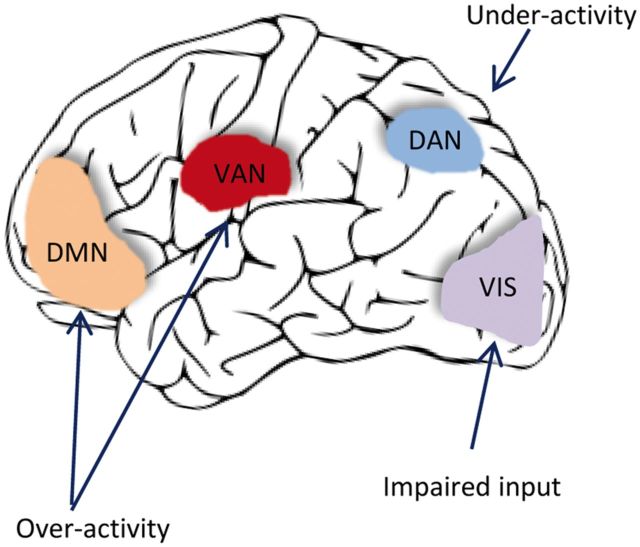
**Theories for visual hallucinations in Parkinson’s disease.** The attention networks hypothesis for visual hallucinations in Parkinson’s disease (Shine *et al.*, 2014). The DAN (involving dorsolateral prefrontal cortex and posterior parietal cortex) is engaged in voluntary orienting; the VAN (involving the lateral and inferior prefrontal cortex and amygdala) engages attention to salient stimuli and mediates activation of other networks; the DMN (involving the medial temporal and medial prefrontal cortex) is engaged in task independent introspective tasks); VIS = visual cortex, processing of visual information. In this model, visual hallucinations are caused by over-reliance on the DMN and the VAN in processing ambiguous percepts, with relative inability to recruit the DAN. Adapted from Shine *et al.* (2015) with permission.

Visual hallucinations in Parkinson’s disease have also been claimed to be due to breakdown in the connectivity in the neural networks subserving attention and conscious perception (Shine *et al.*, 2014) (Fig. 3). These comprise the Dorsal Attention Network (DAN), the Ventral Attention Network (VAN) and the Default Mode Network (DMN). The DAN is activated during tasks requiring exogenous attention. The DMN is associated with internally focused tasks, e.g. mind wandering, whereas the VAN is used for salience monitoring and mediates the switch between the DAN and the DMN. The attentional networks hypothesis predicts that visual hallucinations in Parkinson’s disease are caused by overactivity of the DMN and VAN reinforcing false images that are then unchecked due to failure to engage the DAN. This leads to over reliance on the DMN and VAN that are poorly suited to interpret ambiguous sensory images.

Change in visual perceptual function seems at least necessary, if not sufficient, for complex hallucinations. Visual dysfunction is more common in Parkinson’s disease patients experiencing hallucinations (Gallagher *et al.*, 2011) and in neuroimaging studies, glucose consumption is reduced in occipito-temporal regions in Parkinson’s patients with visual hallucinations compared to those without (Boecker *et al.*, 2007). Executive dysfunction may also have a role in mediating visual hallucinations: Parkinson’s patients with hallucinations perform worse on tests of executive function compared to those without visual hallucinations (Grossi *et al.*, 2005; Barnes and Boubert, 2008; Shin *et al.*, 2012) and higher cortical Lewy body density is seen in the middle frontal gyrus in patients with visual hallucinations (Harding *et al.*, 2002; Papapetropoulos *et al.*, 2006). Structural neuroimaging shows grey matter atrophy in the medial frontal lobe in Parkinson’s disease patients with visual hallucinations compared to those without (Ramirez-Ruiz *et al.*, 2007; Goldman *et al.*, 2014) as well as in visual processing regions. Hallucinations are difficult to capture using functional neuroimaging due to their transient nature, but a recent study has shown increased activation in frontal regions and decreased activation in visual cortex (Goetz *et al.*, 2014), although no correction for multiple comparisons was reported, making the data difficult to interpret. The prevailing models could provide a framework for the conjunction of visual perceptual deficits with frontal executive dysfunction seen in patients with Parkinson’s disease who experience complex hallucinations.

### Drug modulation of visual symptoms

Parkinson’s drugs can improve ophthalmic visual processing. l-DOPA enhances colour vision and contrast sensitivity in Parkinson’s disease (Bulens *et al.*, 1987; Buttner *et al.*, 1994) and abnormal visual evoked potentials in patients with Parkinson’s disease improve with l-DOPA (Bodis-Wollner and Yahr, 1978), supporting a dopaminergic basis for these deficits in the lower visual pathways. On the other hand, the effects of l-DOPA on higher visual function are less well documented. Emotional face recognition improves with l-DOPA (Sprengelmeyer *et al.*, 2003), but effects of l-DOPA or other Parkinson’s drugs, on other aspects of cortical visual processing are not known.

The effects of Parkinsonian drugs on visual hallucinations associated with Parkinson’s disease are also not fully established. Early reports correlated the incidence of visual hallucinations with dose and duration of treatment with l-DOPA (Sweet *et al.*, 1976; Weiner *et al.*, 1980). However, recent reports are more equivocal. Some groups found no difference in l-DOPA dose or duration between patients with Parkinson’s disease with and without hallucinations (Diederich *et al.*, 1998). Off period visual hallucinations are a recognized, if rare, phenomenon in l-DOPA-treated patients with motor fluctuations (Steiger *et al.*, 1991). Others found that while patients with hallucinations were on a slightly higher daily dose of l-DOPA, their levodopa equivalent dose was no different (Fenelon *et al.*, 2000). A recent study reported that a higher proportion of patients with visual hallucinations were taking l-DOPA (Gallagher *et al.*, 2011) and no association was found with other parkinsonian medications. However, double-blind controlled trials with dopamine agonists consistently show a higher rate of hallucinations than on placebo or l-DOPA (Poewe, 2003; Baker *et al.*, 2009; Bonuccelli *et al.*, 2009; Kulisevsky and Pagonabarraga, 2010; Zhou *et al.*, 2014). The conventional approach to treatment of visual hallucinations reflects this by slowly withdrawing dopamine agonists (Connolly and Lang, 2014). Anticholinergic drugs are a well-recognized cause of visual hallucinations and delirium in elderly patients with Parkinson’s disease (Goetz *et al.*, 1982). Amantadine is also hallucinogenic and both drugs should be withdrawn when hallucinations occur (Postma and Van, 1975).

### Impact on driving

There is a relation between contrast sensitivity and driving performance in Parkinson’s disease and between visual perception and driving ability, particularly in tests measuring visual attention, motion perception and visuospatial construction (Uc *et al.*, 2006, 2009; Devos *et al.*, 2013; for a review see Crizzle *et al.*, 2012). The postural instability and gait disorder phenotype is predictive of poorer on-road driving performance, beyond motor severity scores (Devos *et al.*, 2013), consistent with the association of visuo-perceptual deficits with this phenotype. Although visuo-perceptual tests are useful predictors of driving ability, there is insufficient evidence for them to be used alone to determine driver safety. General advice is to consider visuo-perceptual ability and if there is any doubt, to refer the patient for a formal driving assessment via the national driving authority (Crizzle *et al.*, 2012).

## The role of visuo-perceptual changes as a marker for disease

### Visuo-perceptual measures as diagnostic markers of Parkinson’s disease

Changes in visual performance may be a sensitive marker of Parkinson’s disease. In one study, deficits in colour and contrast sensitivity were shown to have better discriminatory power for early diagnosis of Parkinson’s disease than any other non-motor symptom, including hyposmia and sleep disturbance, in patients within 3 years of diagnosis (Diederich *et al.*, 2010). One theory of disease progression in Parkinson’s disease has suggested that the cerebral cortex is not damaged until the later stages of the disease (Braak *et al.*, 2004). Although the exact pathological correlates of colour and contrast sensitivity changes are unclear, changes in visual perception at the earliest stages of the disease raise concerns about this notion. It is likely that visual measures will be a useful marker of early disease only in specific subgroups of patients with Parkinson’s disease (such as those with prominent early cognitive involvement). Visuo-perceptual deficits (unlike oculomotor changes) are rarely reported in atypical forms of parkinsonism such as progressive supranuclear palsy, multiple system atrophy or corticobasal syndrome. Visual processing abnormalities may prove helpful in differentiating these other diseases from idiopathic Parkinson’s disease.

### Visuo-perceptual measures as predictors of dementia in Parkinson’s disease

Visual function may be important in predicting dementia in Parkinson’s disease. In a prospective study, abnormal colour vision at baseline tripled the odds of developing dementia (Anang *et al.*, 2014). In newly diagnosed patients with Parkinson’s disease, impaired pentagon copying at diagnosis was a strong predictor for development of dementia at 5- (Williams-Gray *et al.*, 2009) and 10-year follow-up (Williams-Gray *et al.*, 2013) and pentagon copying deficits show power to predict dementia even at 2-year follow-up (Kaul and Elble, 2014). Similarly, patients with new multi-domain dementia at 3-year follow-up showed impaired performance in an overlapping figures test at baseline (Shoji *et al.*, 2014). These findings are supported by functional neuroimaging findings: in a longitudinal FDG-PET study of Parkinson’s disease, patients who developed dementia, showed occipito-parietal hypometabolism at baseline (Bohnen *et al.*, 2011). However, pentagon copying involves multiple cognitive domains including executive function, praxis and motor planning areas, in addition to visuo-perceptual processes, making it difficult to make confident claims regarding the role for visual perception in dementia prediction.

Visual symptoms may also predict outcome. Visual hallucinations are strong predictors for nursing home placement (Goetz and Stebbins, 1993) and have been suggested as a harbinger for the onset of dementia in Parkinson’s disease (Santangelo *et al.*, 2007). In nursing home residents with Parkinson’s disease, the presence of visual problems is a predictor of mortality (with age, cognitive impairment and pressure ulcers also predictive) (Fernandez and Lapane, 2002). Identifying the presence of visuo-perceptual deficits may therefore have a critical role in stratifying patients in the clinic for early treatment with cholinesterase inhibitors and for defining enriched populations for disease-modifying interventions in clinical trials aimed at preventing dementia in Parkinson’s disease.

### Optical coherence tomography for measuring disease presence and progression

Optical coherence tomography enables high resolution structural imaging of the retina, with measurement of all retinal layers, *in vivo*. Recent technical advances have led to increasing interest in the role of the retina in monitoring disease activity in Parkinson’s disease (Bodis-Wollner *et al.*, 2014*b*; Lee *et al.*, 2014*a*). The nerve fibre layer is thinned in Parkinson’s disease (Inzelberg *et al.*, 2004; Altintas *et al.*, 2008) and at the macula, thinning is found in the inner retinal layer (Hajee *et al.*, 2009; Shrier *et al.*, 2012). Interocular differences are seen in retinal thickness in Parkinson’s disease (Shrier *et al.*, 2012) with more thinning contralateral to the side of motor symptoms (La Morgia *et al.*, 2013), consistent with an asymmetric process involving the eye on the same side as the affected substantia nigra. Recently, retinal morphology, measured using optical coherence tomography, combined with visual electrophysiology was shown to have high diagnostic yield in Parkinson’s disease (Miri *et al.*, 2016). A correlation between retinal thickness, disease severity (Altintas *et al.*, 2008; Miri *et al.*, 2015) and duration (Miri *et al.*, 2015; Sari *et al.*, 2015) has been reported in some but not all studies (La Morgia *et al.*, 2013). In Parkinson’s disease dementia, there is a correlation between retinal thickness and Mini-Mental State Examination scores (Moreno-Ramos *et al.*, 2013), although this is not found in patients without dementia (Garcia-Martin *et al.*, 2012). Whether this reflects insensitivity of the Mini-Mental State Examination, or an absence of relationship between dementia and retinal thickness is not yet known. Intriguingly, retinal thinning is associated with presence of minor hallucinations (Lee *et al.*, 2014*b*), although this was not found for patients with complex visual hallucinations, consistent with the involvement of higher cognitive domains in these hallucinations. Optical coherence tomography therefore shows promise as a potential biomarker for presence of disease in Parkinson’s disease, but more studies will be needed to assess the role of the retina in predicting dementia in Parkinson’s disease.

### Neuroimaging signatures of visual dysfunction as a marker of disease in Parkinson’s disease

Structural and functional neuroimaging studies are consistent with early involvement of cortical visual processing regions in Parkinson’s disease. Cortical thinning in the occipital cortex, measured using MRI, correlates with disease duration (Jubault *et al.*, 2011), with maximal cortical thinning seen in the lateral occipital complex and parietal regions (Pereira *et al.*, 2012). Metabolic deficits have been reported using PET in occipital cortex (Eberling *et al.*, 1994; Bohnen *et al.*, 1999) and occipital and parietal hypoperfusion in Parkinson’s disease can be detected by measuring regional cerebral blood flow (Abe *et al.*, 2003), that worsens with disease progression (Nagamachi *et al.*, 2008). Arterial spin labelled perfusion MRI, a non-invasive measure of perfusion, similarly shows regions of perfusion deficit in early stages of Parkinson’s disease affecting frontal, parietal and also occipital brain regions (Fernandez-Seara *et al.*, 2012).

Recently, network approaches have been applied to structural and functional MRI data in Parkinson’s disease. These reveal changes in patterns of connectivity between brain regions and strongly implicate early involvement of visual processing regions in Parkinson’s disease. Reduced connectivity is seen in temporo-occipital regions, using resting state functional MRI in patients within 3 years of diagnosis, with connectivity losses strongly correlated with reduced visuospatial performance (Luo *et al.*, 2015). In patients at mid-stage Parkinson’s disease, resting state functional MRI network analysis shows connectivity changes especially affecting visual regions (Gottlich *et al.*, 2013). In Parkinson’s disease patients with mild cognitive impairment, the efficiency of connections in parietal, as well as frontal regions is reduced (Pereira *et al.*, 2015), consistent with involvement of visuo-perceptual processing brain regions in the early stages of dementia in Parkinson’s disease.

## Conclusion and future directions

Alterations in visual function from the retina to higher cortical brain regions have been found in Parkinson’s disease, with some aspects of visuo-perceptual processing worsening with disease progression (Diederich *et al.*, 2002; Gullett *et al.*, 2013) and there are emerging data showing impaired pentagon copying as one of the earliest cognitive deficits in Parkinson’s disease. There are, however, no studies of the power of other markers of visuo-perpetual function to predict dementia in Parkinson’s disease. Further studies examining visuo-perceptual function in Parkinson’s disease are required to correlate performance with other disease features such as laterality of motor symptoms and with clinical phenotypes such as postural instability or tremor predominance. Visuo-perceptual performance also needs to be correlated with common Parkinson’s disease-associated genetic mutations to gain insights into pathophysiological pathways underlying deficits; and with neuroimaging to link visuo-perceptual impairment with anatomical regions. These future combined approaches should enhance understanding of the mechanisms underlying progression of Parkinson’s disease to involve cognition.

## Funding

R.S.W. is supported by a UCL Excellence Fellowship, a grant from the Academy of Medical Sciences and received funding from the department of health’s NIHR Biomedical Research Centre funding scheme (UCL/UCLH). J.D.W. is supported by a Wellcome Senior Clinical Fellowship: grant no. 091673/Z/10/Z. S.J.C. is supported by grants from ESRC/NIHR (ES/L001810/1), EPSRC (EP/M006093/1) and an Alzheimer’s Research UK Senior Research Fellowship. H.R.M. has been supported by Parkinson’s UK (grants 8047, J-0804) and the Medical Research Council (G0700943)

## Abbreviations

DAN: dorsal attention network
DMN: default mode network
RBD: REM sleep behaviour disorder
REM: rapid eye movement
V1: primary visual cortex
VAN: ventral attention network
